# First report of a Mexican family with mutation in the *CDH1* gene

**DOI:** 10.1002/mgg3.1208

**Published:** 2020-09-04

**Authors:** Carmen Martínez Valenzuela, Edmundo Erbey Castelán‐Maldonado, Octavio Carvajal‐Zarrabal, Ana Laura Calderón‐Garcidueñas

**Affiliations:** ^1^ Unidad de Investigación en ambiente y salud Universidad Autónoma de Occidente Los Mochis Sinaloa México; ^2^ Hospital de Especialidades UMAE 25 Instituto Mexicano del Seguro Social Monterrey NL México; ^3^ Facultad de Medicina Veracruz México

**Keywords:** breast carcinoma, *CDH1* gene, gastric carcinoma, Mexican family

## Abstract

**Background:**

Germline mutations in E‐cadherin (*CDH1*) gene are associated with autosomal‐dominantly inherited cancer syndrome characterized by diffuse gastric cancer, lobular breast cancer, and in some families, cleft lip/palate. However, there may be generations in which these neoplasms do not occur at all in a family and later on, one or another carcinoma arises, which makes it difficult for physicians to think about hereditary origin.

**Methods:**

We report the first Mexican family with *CDH1* mutation (variant c.377del).

**Results:**

An asymptomatic young woman underwent a search for mutations in susceptibility genes for breast cancer due to the history of this neoplasm in her mother and maternal aunt. A *CDH1* mutation was detected. After an endoscopy, a diffuse gastric carcinoma was found. Later on, three generations of this family were studied. The findings are presented.

**Conclusion:**

Medical communities should be aware of the contribution of this gene in the development of hereditary diffuse gastric carcinoma (HDGC) and breast cancer.

## Graphical abstract

First report of a Mexican family with mutation in the CDH1 gene. CDH1 mutation (variant c.377del). There may be generations in which neoplasms do not occur at all in a family and later on, breast or gastric carcinoma arises, which makes it difficult for physicians to think about hereditary origin.

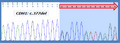

## 1 INTRODUCTION

Germline mutations in E‐cadherin gene (MIM 192,090; *CDH1*) are associated with autosomal‐dominantly inherited cancer syndrome characterized by hereditary diffuse gastric cancer (HDGC), lobular breast cancer (BC) (Hansford et al., 2015), and in some families, cleft lip/palate (Obermair et al., 2019).

The cumulative incidence of gastric cancer by the age of 80 years is around 56% (95% CI, 44%–69%) for women and 70% (95% CI, 59%–80%) for men, while the risk of BC for women is 42% (95% CI, 23%–68%) (Hansford et al., 2015).

The tumor suppressor gene *CDH1* located on chromosome 16q22 has 16 exons (100 kb) and codes for E‐cadherin protein (Gall & Frampton, 2013). This transmembrane glycoprotein is responsible for cell adhesion and invasion‐suppression functions through Ca^2+‐^binding regions in its extracellular domain (Gall & Frampton, 2013).

Tissue integrity requires a functional adherens junction (AJ) (Bianchini et al., 2015). Formation of the AJ is directed by the cadherin–β catenin complex (CCC); αE‐catenin links the CCC to F‐actin (Bianchini et al., 2015). The normal development of the head and face (craniofacial development), including the eyelids and teeth, depends on the interactions between the E‐cadherin and p120‐catenin proteins. E‐cadherin is also a tumor suppressor protein (Zhang et al., 2018). One to three percent of gastric cancer patients have HDGC whose main cause is a germline mutation in the *CDH1* gene in 30%–50% of cases (Fitzgerald et al., 2010). There are at least 100 different germline mutations of *CDH1,* especially frameshift mutations that cause a truncated protein; also exon/intron splice site mutations and single nucleotide mutations have been described. Large exonic deletions account for 4% of these mutations (Fitzgerald et al., 2010). Although there is a study about E‐cadherin expression in sporadic gastric cancer from Mexico (Gamboa‐Dominguez et al., 2015), there are no reports of families with germline mutations in this gene. We report the first Mexican family with *CDH1* mutation in which three generations are studied.

## 2 MATERIAL AND METHODS

Editorial Policies and Ethical Considerations: The study was approved by the Ethics Committee and the participants signed an informed consent.

### 2.1 Family report

**Figure 1:**
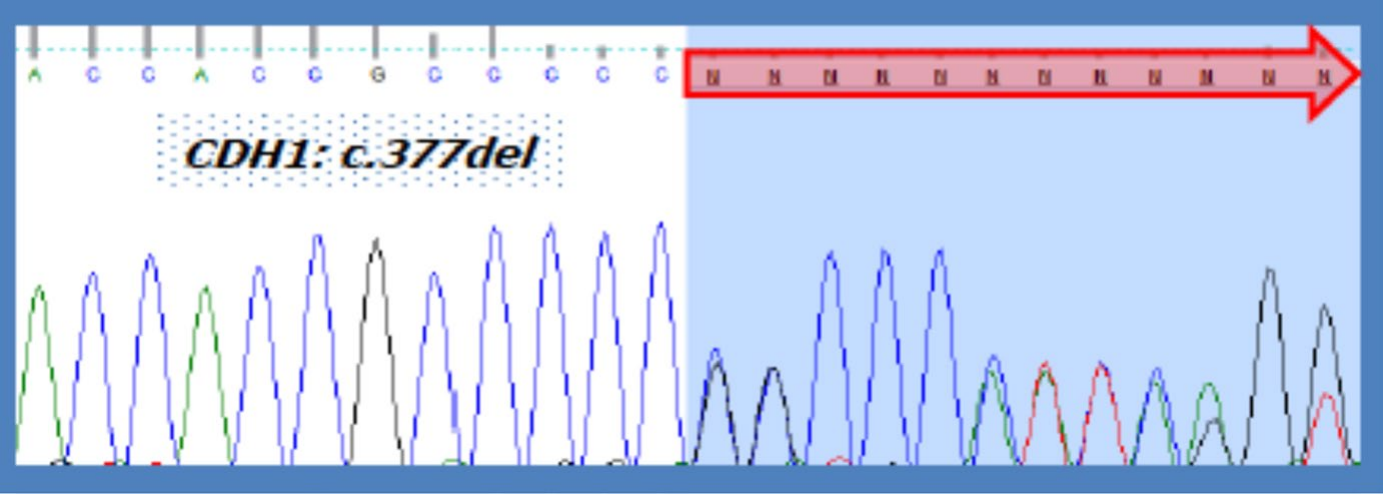
Partial electropherogram of CDH1 gene sequencing (HGNC:1748; LRG_301t1; ENSG00000039068.19). The arrow shows affectation on the reading frame sequence in the allele affected by variant c.377del (p.Pro126Argfs * 89)

**Figure 2:**
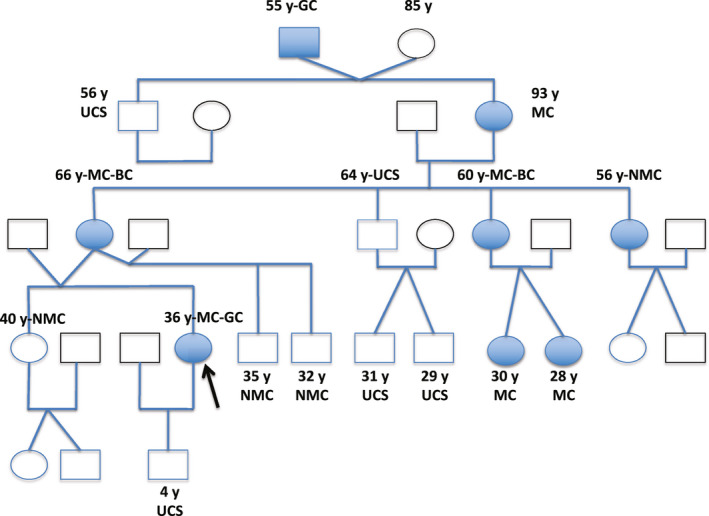
Genealogical tree of the family with the mutation c.377del (p.Prol126Argfs * 89) in the *CDH1* gene. BC, breast cancer; circle, female; GC, Gastric carcinoma; MC, mutation carrier; NMC, no mutation carrier; square, male; UCS, unknown carrier status; y, years old

A 35‐year‐old woman underwent multigene panel testing in genes associated with BC due to the history of this neoplasm in her mother and a maternal aunt who presented the tumor at 55 and 50 years of age, respectively. The study ruled out mutations in the following genes*: BRCA1* (MIM 113705*), BRCA2* (MIM 600185)*, TP53* (MIM 191170), *PTEN* (MIM 601728), and *STK11* (MIM 602216)*;* however, the variant c.377delC [NM_004360.5(CDH1):c.377del (p.Pro126Argfs* 89); LOVD, Genomic variant #0000058778; individual ID 00032542] in heterozygous state within the sequence of the *CDH1* gene (HGNC:1748; LRG_301t1; ENSG00000039068.19) was identified (Figure 1). The young woman underwent a gastric endoscopy that showed no macroscopic alterations, however, the biopsies showed an undifferentiated diffuse adenocarcinoma with signet‐ring cells. The patient underwent a total gastrectomy and Roux‐en‐Y esophagojejunostomy. The definitive histopathological study confirmed the presence of intramucosal early gastric adenocarcinoma. Given the molecular findings, other members of the family were analyzed for this specific mutation. The results are shown in Figure 2.

The patient had several postoperative complications including transient dysphagia and esophageal stenosis that required dilatations. The relatives carrying the mutation are for the time being in endoscopic follow‐up with annual gastric biopsies; breast MRI and breast ultrasound are performed in those women under 40 years of age.

## 3 DISCUSSION

Since the first report of *CDH1* germline mutations in a Maori population from New Zealand with a strong cluster for gastric tumors (Jones, 1964), more information about this gene has been collected. Later on, the association of gastric cancer and lobular breast carcinoma in families with mutations in the *CDH1* gene was reported (Keller et al., 1999). Therefore, these two entities have been part of the HDGC syndrome (Corso et al., 2018). However, there may be generations in which these neoplasms do not occur at all, in the same family and, later on, one type or another of neoplasm arises, which makes it difficult for physicians to think about the possibility of a hereditary case. Such is the situation of the family reported. The index case of gastric carcinoma, a young woman, 35 years old, was screened for mutations due to a history of BC in her mother and aunt who presented the neoplasm between 50 and 55 years of age. After the finding of the mutation, the mother, one sister, two aunts, two cousins, and the maternal grandmother were tracked for the mutation. The maternal great‐grandfather had died from a DGC with signet ring cells at 55 years of age. Initially, in this man it was considered that having blood group A and being a heavy smoker had contributed to the development of cancer. However, in retrospect it was evident that the mutation in the index case came from the mother's line. It calls the attention that the maternal grandmother (mutation carrier) died at 93 years of age due to cardiac involvement and the development of neoplasia was never documented. Her last mammography, at 85 years old of age, showed a fatty mammary pattern, BIRADS 1. The two confirmed BC cases were a lobular carcinoma and a moderately differentiated ductal carcinoma, both with positive estrogen and progesterone receptors; they had a bilateral mastectomy and chemotherapy and are currently asymptomatic.

With *CDH1* mutations, the cumulative risk of developing DGC by age 80 is 70 and 56% in men and women, respectively. The cumulative risk for lobular BC is of 42% in women by age 80 (Hansford et al., 2015). In the other hand, somatic CDH1 mutations are detected in 10%–56% of lobular BC and in 5% of ductal carcinomas (Lei et al., 2002).

In Western medicine there seems to be consensus about prophylactic total gastrectomy in patients with germline mutation in the *CDH1* gene; surgery is usually performed in 18‐ to 40‐year‐old carriers (van der Post et al., 2015). Foci of intramucosal signet ring cell carcinomas have been observed in 80%–83% of the resected stomachs (Gjyshi et al., 2018; Pantelis et al., 2018), even in patients of 14 years of age (Gullo et al., 2018). However, prophylactic total gastrectomy has considerable morbidity and functional consequences. Understanding the pathophysiology of the damage produced by mutations would help to propose new treatments.

Human E‐cadherin is a 120‐kDa transmembrane glycoprotein; the extracellular component has five repeat domains with four calcium‐binding sites that are important in stabilization of the conformation; the membrane component connects the extracellular part of E‐cadherin to the cytoplasmic part, containing the recognition sites for catenins (Kellen, 1996). This cytoplasmic part binds to beta‐ or gamma‐catenin and to p120^cas^, and also to alpha‐catenin. Alpha‐catenin forms the link with α‐actinin and F‐actin. The downregulation of E‐cadherin in malignant mammary cells is associated with expression of vimentin for invasion to occur. Experimental studies have shown that tamoxifen activates the E‐cadherin/catenin complex. In fact, insulin‐like growth factor 1, retinoic acid, and the citrus flavonoid tangeretin can activate the E‐cadherin/catenin complex (Kellen, 1996) and this complex could be a therapeutic target.

In the case of families with hereditary DGC, germline mutations of *CDH1* gene occur in 30% of the cases; of these, 23% are missense mutations. Truncating *CDH1* mutations are the majority of *CDH1* germline variants followed by missense variants, and variants affecting splice sites [Kellen, 1996]. Mutations affect each of the 16 exons of the gene, with some hotspots for truncating mutations c.1003C>T, c.1212delC, c.1137G>A, 1792C>T, or 2398delC [Li, Lo, & Rudloff, 2018).

Mutations in *CDH1* gene also may be associated with colon cancer. In fact, when 1,000 patients with advanced cancer were studied using exome sequencing of a 202‐gene panel, 151 patients showed germline variants and 15 patients had pathogenic germline variants, including two cases with *CDH1* mutations (You et al., 2019). It is reported that families with truncating variants in the PRE‐PRO region have an increased probability of colorectal cancer, when compared with variants in other regions (Lo et al., 2019).

In the other hand, somatic mutations occur in both sporadic and hereditary forms. In case of HDGC not associated with *CDH1* germline mutations, promoter hypermethylation has been described in 50% of patients (Corso et al., 2013). The methylation status in subjects with mutations in the germline of the *CDH1* gene could also contribute to explain the phenotypic variability in the different generations.

It is logical to think that mutations affecting different E‐cadherin protein domains have distinct effects in motility. Experiments in cell lines with different *CDH1* missense mutations localized on the extracellular and juxtamembrane domains, both affecting the integrity of the extracellular domain, led to increased cell motility accompanied by increased epidermal growth factor receptor (EGFR) activation, and also increased activation of Src kinase and p38 mitogen‐activated protein kinases (MAPK). In this type of mutations there seems to be a genotype–phenotype correlation that defines a subset of HDGC cases which may benefit from EGFR inhibitors (Mateus et al., 2009).

Experiments with cancer rat model using NMU (N‐nitroso‐N‐metilurea) as carcinogen had shown that 9‐cis‐isomer of retinoic acid (9cRA) is effective for preventing mammary cancer and enhances the chemopreventive activity of low doses of tamoxifen, increasing expression of E‐ cadherine, at cell–cell contact sites in BC cells. These results suggest that retinoids and tamoxifen may be useful not only in the prevention of breast cancer but also in its treatment (Anzano et al., 1994).

The recommended prophylactic treatment for carriers of germline mutations in the *CDH1* gene is total gastrectomy (van der Post et al., 2015; Strong et al., 2017); bloc resection of gastric mucosa in early carcinoma is an accepted treatment in those patients who are not carriers of mutations, however, it is not enough in patients with germline mutations as the neoplasm can develop anywhere in the mucosa. Perfecting techniques like mucosal (EMR) and endoscopic submucosal dissection (ESD) combined with new hemostat agents for exudative hemorrhages from small vessels in the gastrointestinal tract could be an option (Subramaniam, Kandiah, Thayalasekaran, Longcroft‐Wheaton, & Bhandari, 2019).

Medical communities should be aware of the contribution of this gene in the development of gastric carcinoma, mammary carcinoma, and occasionally colon cancer.

## CONFLICTS OF INTEREST

The authors declare that there are no conflicts of interest.

## References

[mgg31208-bib-0001] Anzano, M. A. , Byers, S. W. , Smith, J. M. , Peer, C. W. , Mullen, L. T. , Brown, C. C. , … Sporn, M. B. (1994). Prevention of breast cancer in the rat with 9‐cis‐retinoic acid as a single agent and in combination with tamoxifen. Cancer Research, 54, 4614–4617. 8062253

[mgg31208-bib-0002] Bianchini, J. M. , Kitt, K. N. , Gloerich, M. , Pokutta, S. , Weis, W. I. , & Nelson, W. J. (2015). Reevaluating αE‐catenin monomer and homodimer functions by characterizing E‐cadherin/αE‐catenin chimeras. Journal of Cell Biology, 210, 1065–1074. 10.1083/jcb.201411080 26416960 PMC4586751

[mgg31208-bib-0003] Corso, G. , Carvalho, J. , Marrelli, D. , Vindigni, C. , Carvalho, B. , Seruca, R. , … Oliveira, C. (2013). Somatic mutations and deletions of the E‐cadherin gene predict poor survival of patients with gastric cancer. Journal of Clinical Oncology, 31, 868–875. 10.1200/JCO.2012.44.4612 23341533

[mgg31208-bib-0004] Corso, G. , Figueiredo, J. , La Vecchia, C. , Veronesi, P. , Pravettoni, G. , Macis, D. , … Galimberti, V. (2018). Hereditary lobular breast cancer with an emphasis on E‐cadherin genetic defect. Journal of Medical Genetics, 55, 431–441. 10.1136/jmedgenet-2018-105337 29929997

[mgg31208-bib-0005] Fitzgerald, R. C. , Hardwick, R. , Huntsman, D. , Carneiro, F. , Guilford, P. , Blair, V. , & … International Gastric Cancer Linkage Consortium (2010). Hereditary diffuse gastric cancer: Updated consensus guidelines for clinical management and directions for future research. Journal of Medical Genetics, 47, 436–444. 10.1136/jmg.2009.074237 20591882 PMC2991043

[mgg31208-bib-0006] Gall, T. M. , & Frampton, A. E. (2013). Gene of the month: E‐cadherin (*CDH1*). Journal of Clinical Pathology, 66, 928–932. 10.1136/jclinpath-2013-201768 23940132

[mgg31208-bib-0007] Gamboa‐Dominguez, A. , Dominguez‐Fonseca, C. , Chavarri‐Guerra, Y. , Vargas, R. , Reyes‐Gutierrez, E. , Green, D. , & Quintanilla‐Martinez, L. (2015). E‐cadherin expression in sporadic gastric cancer from Mexico: Exon 8 and 9 deletions are infrequent events associated with poor survival. Human Pathology, 36, 9–35. 10.1016/j.humpath.2004.09.020 15712179

[mgg31208-bib-0008] Gjyshi, O. , Vashi, P. , Seewald, L. , Kohan, M. , Abboud, E. , Fowler, E. , … Halabi, H. (2018). Therapeutic and prophylactic gastrectomy in a family with hereditary diffuse gastric cancer secondary to a *CDH1* mutation: A case series. World Journal of Surgical Oncology, 16, 143 10.1186/s12957-018-1415-5 30007404 PMC6046101

[mgg31208-bib-0009] Gullo, I. , Devezas, V. , Baptista, M. , Garrido, L. , Castedo, S. , Morais, R. , … Carneiro, F. (2018). Phenotypic heterogeneity of hereditary diffuse gastric cancer: Report of a family with early‐onset disease. Gastrointestinal Endoscopy, 87, 1566–1575. 10.1016/j.gie.2018.02.008 29454568

[mgg31208-bib-0010] Hansford, S. , Kaurah, P. , Li‐Chang, H. , Woo, M. , Senz, J. , Pinheiro, H. , … Huntsman, D. G. (2015). Hereditary diffuse gastric cancer syndrome: *CDH1* mutations and beyond. JAMA Oncology, 1, 23–32. 10.1001/jamaoncol.2014.168 26182300

[mgg31208-bib-0011] Jones, E. G. (1964). Familial gastric cancer. NZ. Med. J., 63, 287–296. 14158754

[mgg31208-bib-0012] Kellen, J. A. (1996). Tamoxifen beyond the antiestrogen. Boston: Birkhäuser Basel.

[mgg31208-bib-0013] Keller, G. , Vogelsang, H. , Becker, I. , Hutter, J. , Ott, K. , Candidus, S. , … Höfler, H. (1999). Diffuse type gastric and lobular breast carcinoma in a familial gastric cancer patient with an E‐cadherin germline mutation. American Journal of Pathology, 155, 337–342. 10433926 10.1016/S0002-9440(10)65129-2 PMC1866861

[mgg31208-bib-0014] Lei, H. , Sjöberg‐Margolin, S. , Salahshor, S. , Werelius, B. , Jandáková, E. , Hemminki, K. , & Lindblom, A. (2002). *CDH1* mutations are present in both ductal and lobular breast cancer, but promoter allelic variants show no detectable breast cancer risk. International Journal of Cancer, 98, 199–204. 11857408 10.1002/ijc.10176

[mgg31208-bib-0015] Li, D. , Lo, W. , & Rudloff, U. (2018). Merging perspectives: Genotype‐directed molecular therapy for hereditary diffuse gastric cancer (HDGC) and E‐cadherin‐EGFR crosstalk. Clinical and Translational Medicine, 7, 7 10.1186/s40169-018-0184-7 29468433 PMC5821620

[mgg31208-bib-0016] Lo, W. , Zhu, B. , Sabesan, A. , Wu, H.‐H. , Powers, A. , Sorber, R. A. , … Rudloff, U. (2019). Associations of CDH1 germline variant location and cancer phenotype in families with hereditary diffuse gastric cancer (HDGC). Journal of Medical Genetics, 56, 370–379. 10.1136/jmedgenet-2018-105361 30745422 PMC6716162

[mgg31208-bib-0017] Mateus, A. R. , Simões‐Correia, J. , Figueiredo, J. , Heindl, S. , Alves, C. C. , Suriano, G. , & Luber, B. (2009). E‐cadherin mutationand cell motility: A genotype‐phenotype correlation. Experimental Cell Research, 315, 1393–1402. 10.1016/j.yexcr.2009.02.020 19268661

[mgg31208-bib-0018] Obermair, F. , Rammer, M. , Burghofer, J. , Malli, T. , Schossig, A. , Wimmer, K. , & Kranewitter, W. (2019). Cleft lip/palate and hereditary diffuse gastric cancer: Report of a family harboring a *CDH1* c.687 + 1G > A germline mutation and review of the literature. Familial Cancer, 18, 253–260. 10.1007/s10689-018-0111-5 30306390

[mgg31208-bib-0019] Pantelis, D. , Lingohr, P. , Hueneburg, R. , & Spie, R. J. , Vilz, T. , Lau, J. F. , & Nattermann, J. (2018). Outcomes after prophylactic total gastrectomy for hereditary diffuse gastric cancer. Zentralblatt Fur Chirurgie, 10.1055/a-0646-4382 30068014

[mgg31208-bib-0020] Strong, V. E. , Gholami, S. , Shah, M. A. , Tang, L. H. , Janjigian, Y. Y. , Schattner, M. , … Coit, D. G. (2017). Total gastrectomy for hereditary diffuse gastric cancer at a single center: Postsurgical outcomes in 41 patients. Annals of Surgery, 266, 1006–1012. 10.1097/SLA.0000000000002030 27759617

[mgg31208-bib-0021] Subramaniam, S. , Kandiah, K. , Thayalasekaran, S. , Longcroft‐Wheaton, G. , & Bhandari, P. (2019). Haemostasis and prevention of bleeding related to ER: The role of a novel self‐assembling peptide. United European Gastroenterology Journal, 7, 155–162. 10.1177/2050640618811504 30788128 PMC6374844

[mgg31208-bib-0022] van der Post, R. S. , Vogelaar, I. P. , Carneiro, F. , Guilford, P. , Huntsman, D. , Hoogerbrugge, N. , & Caldas, C. (2015). Hereditary diffuse gastric cancer: Updated clinical guidelines with an emphasis on germline *CDH1* mutation carriers. Journal of Medical Genetics, 52, 361–374. 10.1136/jmedgenet-2015-103094 25979631 PMC4453626

[mgg31208-bib-0023] You, Y. N. , Borras, E. , Chang, K. , Price, B. A. , Mork, M. , Chang, G. J. , … Vilar, E. (2019). Detection of pathogenic germline variants among patients with advanced colorectal cancer undergoing tumor genomic profiling for precision medicine. Diseases of the Colon and Rectum, 62, 429–437. 10.1097/DCR.0000000000001322 30730459 PMC6415928

[mgg31208-bib-0024] Zhang, H. , Feng, M. , Feng, Y. , Bu, Z. , Li, Z. , Jia, S. , & Ji, J. (2018). Germline mutations in hereditary diffuse gastric cancer. Chinese Journal of Cancer Research, 30, 122–130. 10.21147/j.issn.1000-9604.2018.01.13 29545726 PMC5842226

